# Effect of UV-A Irradiation on Bioactive Compounds Accumulation and Hypoglycemia-Related Enzymes Activities of Broccoli and Radish Sprouts

**DOI:** 10.3390/plants13030450

**Published:** 2024-02-03

**Authors:** Gongheng Che, Mingmei Chen, Xiaodan Li, Junxia Xiao, Liang Liu, Liping Guo

**Affiliations:** 1 College of Food Science and Engineering, Qingdao Agricultural University, Qingdao 266109, China; chegongheng1204@163.com (G.C.); cmm1954154242@163.com (M.C.); lixd1231@163.com (X.L.); xjxfood@qau.edu.cn (J.X.); 201901104@qau.edu.cn (L.L.); 2 Key Laboratory of Special Food Processing (Co-construction by Ministry and Province), Ministry of Agriculture Rural Affairs, Qingdao Agricultural University, Qingdao 266109, China; 3 Shandong Technology Innovation Center of Special Food, Qingdao 266109, China

**Keywords:** UV-A irradiation, bioactive compounds, hypoglycemia-related enzymes, broccoli sprouts, radish sprouts

## Abstract

In the present study, different intensities of UV-A were applied to compare their effects on growth, bioactive compounds and hypoglycemia-related enzyme activities in broccoli and radish sprouts. The growth of sprouts was decreased after UV-A irradiation. A total of 12 W of UV-A irradiation resulted in the highest content of anthocyanin, chlorophyll, polyphenol and ascorbic acid in broccoli and radish sprouts. The highest soluble sugar content was recorded in sprouts under 8 W of UV-A irradiation, while no significant difference was obtained in soluble protein content among different UV-A intensities. Furthermore, 12 W of UV-A irradiation induced the highest glucosinolate accumulation, especially glucoraphanin and glucoraphenin in broccoli and radish sprouts, respectively; thus, it enhanced sulforaphane and sulforaphene formation. The α-amylase, α-glucosidase and pancrelipase inhibitory rates of two kinds of sprouts were enhanced significantly after UV-A irradiation, indicating UV-A-irradiation-treated broccoli and radish sprouts have new prospects as hypoglycemic functional foods.

## 1. Introduction

Cruciferous sprouts are rich in glucosinolates, phenolic compounds, anthocyanins, ascorbic acid, etc., which are related to the prevention of chronic diseases. Especially, broccoli and radish sprouts have attracted extensive attention from researchers due to their various and prominent biological activities. It has been reported that broccoli sprouts are effective against various cancers and novel coronaviruses, as well as possessing antioxidant, anti-inflammatory, antibacterial, analgesic, antidepressant and anti-obesity properties, etc. [1,2,3]. On the other hand, radish sprouts also have positive antioxidant, anti-cancer, anti-inflammatory and anti-radical properties [4,5]. In addition, radish sprouts are thought to have greater anti-cancer potential than broccoli sprouts due to the absence of epithiospecifier protein (ESP), resulting in a higher yield of isothiocyanates with anti-cancer properties [6]. Hence, the enhancement of accumulation of bioactive compounds and bioactivities is important in developing the corresponding functional foods of broccoli and radish sprouts and their industrial production.

Plant photoreceptors detect light with a variety of wavelengths, such as blue (400–500 nm) and ultraviolet (280–315 nm), and mount responses that are specific to the wavelength [7]. Ultraviolet light (UV) as an abiotic stressor, can cause different responses in plants, including damage to DNA and proteins, the generation of reactive oxygen species (ROS), changes in plant growth and development and the enhancing of secondary metabolites [8]. Moreira-Rodríguez et al. [9] reported that UV-A treatment was able to enhance the content of phenolic compounds in broccoli sprouts, such as gallic acid and kaempferol glucoside, compared to UV-B treatment. Kang et al. [8] found that the phenolic compound content and phenylalanine ammonia lyase (PAL) activity of sweet basil sprouts were highest at a UV-A intensity of 20 W·m^−2^, but showed an inhibitory effect at a UV-A intensity of 30 W·m^−2^. The results of Jiang et al. [10] showed that supplementation with 12 µmol·m^−2^ s^−1^ UV-A significantly increased the content of total glucosinolates in kale sprouts; meanwhile, 4-methoxyglucobrassicin and neoglucobrassicin content increased by 31.29% and 79.02%, respectively, compared to the control. Flavonoids are a kind of polyphenols, and anthocyanins are a kind of flavonoids; they are widely found in plants. Studies have shown that polyphenol intake is associated with a reduced risk of chronic disease, and the contents of polyphenol, flavonoid and anthocyanin in “red butter” lettuce sprouts under UV-A treatment were higher than the control with 17.24%, 11.57% and 14.11%, respectively [11]. However, there is limited information on the effects of UV-A irradiation on the enrichment of secondary metabolites of broccoli and radish sprouts.

Since the 21st century, the number of patients with type II diabetes mellitus (T2DM) has increased dramatically, and has become a serious public health problem worldwide. Given the association between diabetes and premature death, effective measures must be taken to prevent it in our daily life. Recently, the nutritional and medicinal values of sprouts have been extensively researched in many countries, and it is generally accepted that sprouts have some potential in the prevention and treatment of diabetes. It has been reported that fava bean sprouts showed the highest improvement effect in lowering blood glucose levels in diabetic rats with a reduction from 399.2 mg/dL to 139.6 mg/dL, as well as reduced complications, including lipid metabolism and oxidative stress associated with diabetes [12]. Additionally, Laila et al. [13] found that fenugreek sprouts exhibited the best α-glucosidase inhibitory activity (95.24%) and also showed a moderate inhibitory effect on α-amylase (41.64%) and invertase (49.24%) at the vitro level. As we all know, broccoli and radish sprouts are rich in phenolic compounds and glucosinolates metabolite, isothiocyanates, which have a blood glucose-lowering effect. It deserves to be investigated whether broccoli and radish sprouts have an effect on lowering blood glucose levels.

Therefore, the aim of the present study was to investigate the growth and the content of secondary metabolites, such as the glucosinolates, isothiocyanates, phenolic compounds, anthocyanins and ascorbic acid of broccoli and radish sprouts under UV-A irradiation, and also to compare the hypoglycemic enzyme activities, including the α-amylase, α-glucosidase and pancrelipase of broccoli and radish sprouts in vitro level.

## 2. Results

### 2.1. Effect of UV-A Irradiation on Growth and Anthocyanin, Chlorophyll Content in Broccoli and Radish Sprouts

**Figure 1:**
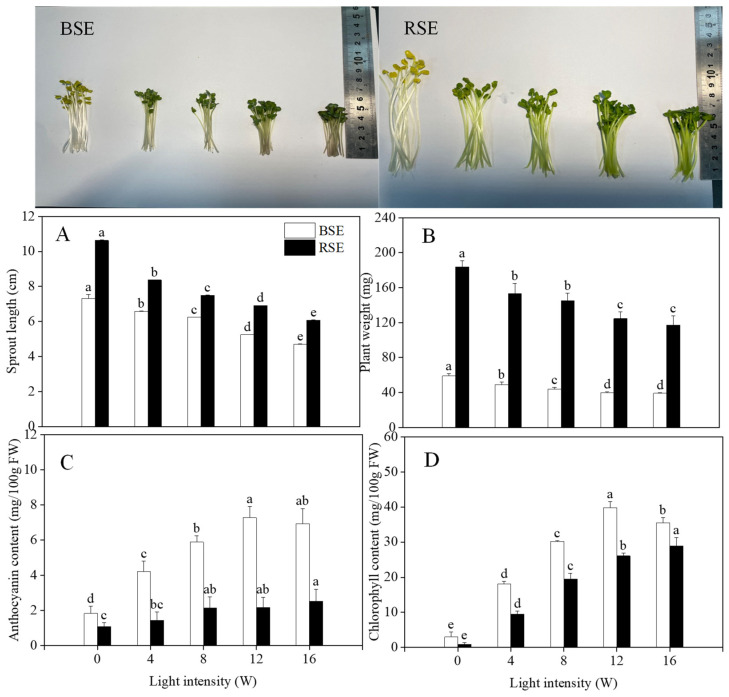
Effect of UV-A irradiation on sprout length (**A**), plant weight (**B**), anthocyanin content (**C**) and chlorophyll content (**D**) in broccoli and radish sprouts. BSE: broccoli sprout extracts; RSE: radish sprout extracts. 0 W: seeds treated with dark; 4 W, 8 W, 12 W, 16 W: seeds treated with UV-A, respectively. The letters of a–e are significantly different at *p <* 0.05.

As shown in Figure 1A,B, UV-A irradiation inhibited the growth of broccoli and radish sprouts. With the increase in light intensity, the length and weight of broccoli and radish sprouts showed different degrees of reduction. At a light intensity of 16 W, the length of the broccoli and radish sprouts were lower than the control by 44.05% and 42.82%, respectively; the plant weight was reduced by 34.19% and 36.30% compared to the control, respectively. From Figure 1C, the highest anthocyanin content was 7.27 mg/100 g at a 12 W light intensity in broccoli sprouts and 2.52 mg/100 g at 16 W light intensity in radish sprouts, which was 3.97 and 2.36 times of that in the control, respectively. In Figure 1D, UV-A irradiation significantly increased the chlorophyll enrichment of both sprouts. The highest chlorophyll content in broccoli sprouts was recorded at 12 W light intensity, which was 13.45 times of control, and showed a decreasing trend at 16 W. The highest chlorophyll content in radish sprouts was recorded at 16 W and was 31.42 times that of the control.

### 2.2. Effect of UV-A Treatment on Soluble Sugar and Soluble Protein Content in Broccoli and Radish Sprouts

**Figure 2:**
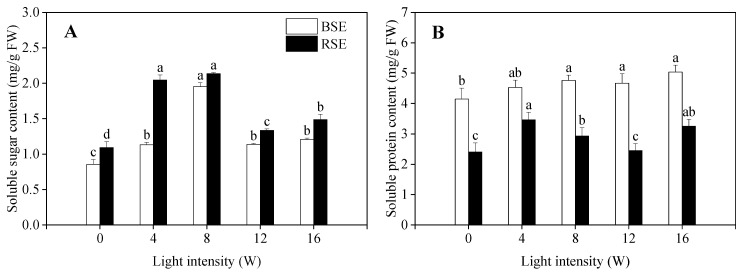
Effect of UV-A irradiation on soluble sugar content (**A**) and soluble protein content (**B**) in broccoli and radish sprouts. BSE: broccoli sprout extracts; RSE: radish sprout extracts. 0 W: seeds treated with dark; 4 W, 8 W, 12 W, 16 W: seeds treated with UV-A, respectively. The letters of a–d are significantly different at *p <* 0.05.

Soluble sugar content in radish sprouts was higher than that in broccoli sprouts, and UV-A irradiation significantly enhanced its content, compared to the control (Figure 2A). A UV-A intensity of 8 W resulted in the highest soluble sugar content in broccoli and radish sprouts, which was 2.27 and 1.96 times of that in the control, respectively. As can be seen from Figure 2B, no significant difference was found in the soluble protein content in broccoli sprouts among different UV-A intensities; however, it first increased and then decreased in radish sprouts with the increasing of UV-A intensity.

### 2.3. Effect of UV-A Irradiation on Polyphenol and Ascorbic Acid Content in Broccoli and Radish Sprouts

**Figure 3:**
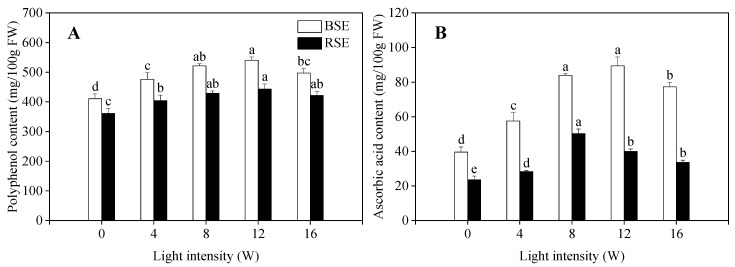
Effect of UV-A irradiation on polyphenol content (**A**) and ascorbic acid content (**B**) in broccoli and radish sprouts. BSE: broccoli sprouts extracts; RSE: radish sprouts extracts. 0 W: seeds treated with dark; 4 W, 8 W, 12 W, 16 W: seeds treated with UV-A respectively. The letters of a–e are significantly different at *p <* 0.05.

As shown in Figure 3, the total polyphenol content and ascorbic acid content in broccoli and radish sprouts was first increased and then decreased with the increasing of UV-A intensity. The polyphenol content in broccoli and radish sprouts was 540.26 and 443.55 mg/100 g at a 12 W light intensity, which was 1.31 and 1.23 times of that in the control samples, respectively (Figure 3A). On the other hand, the highest ascorbic acid content in the broccoli and radish sprouts was recorded at the light intensity of 8 W, which was 2.12 and 2.13 times in comparison with the control, respectively (Figure 3B).

### 2.4. Effect of UV-A Irradiation on Glucosinolate Content in Broccoli and Radish Sprouts

UV-A irradiation significantly induced the accumulation of aliphatic and indole glucosinolates in broccoli and radish sprouts (Table 1). It was found that GRA and RAE, the most abundant glucosinolate in broccoli and radish sprouts, respectively; showed a trend of increasing, then decreasing with the increasing of UV-A intensity, and it reached the highest level at 12 W UV-A intensity, which was 2.99 and 2.84 times that of the control. Moreover, other individual glucosinolates in the broccoli and radish sprouts were also significantly enhanced after UV-A irradiation. The highest total glucosinolate content in broccoli and radish sprouts was found at a 12 W UV-A intensity, which was 2.57 and 2.20 times that of the control.

### 2.5. Effect of UV-A Irradiation on Myrosinase Activity of Broccoli and Radish Sprouts

**Figure 4:**
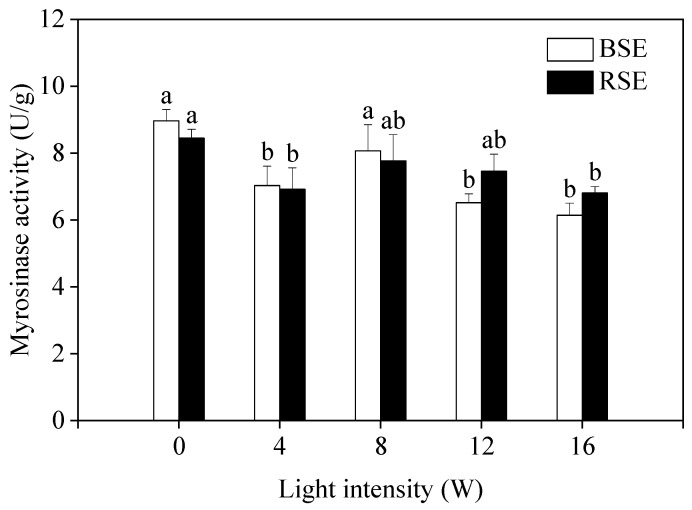
Effect of UV-A irradiation on myrosinase activity in broccoli and radish sprouts. BSE: broccoli sprout extracts; RSE: radish sprout extracts. 0 W: seeds treated with dark; 4 W, 8 W, 12 W, 16 W: seeds treated with UV-A, respectively. The letters of a–b are significantly different at *p <* 0.05.

It was noted that UV-A did not affect reduced myrosinase activity in broccoli and radish sprouts. In addition, no significant difference was observed in myrosinase activity between the two kinds of sprouts. The lowest myrosinase activity was found in two sprouts treated with a UV-A intensity of 16 W, which was decreased by 46.09% and 24.08% compared to the control, respectively (Figure 4).

### 2.6. Effect of UV-A Irradiation on Sulforaphane and Sulforaphene Formation in Broccoli and Radish Sprouts

**Figure 5:**
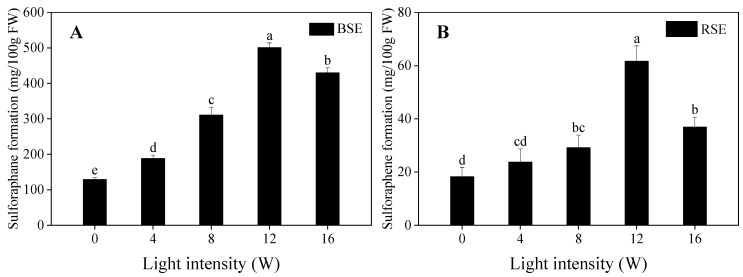
Effect of UV-A irradiation on sulforaphane formation (**A**) and sulforaphane formation (**B**) in broccoli and radish sprouts. BSE: broccoli sprout extracts; RSE: radish sprout extracts. 0 W: seeds treated with dark; 4 W, 8 W, 12 W, 16 W: seeds treated with UV-A, respectively. The letters of a–e are significantly different at *p <* 0.05.

As can be seen from Figure 5A,B, all UV irradiation significantly induced sulforaphane and sulforaphene formation in broccoli and radish sprouts compared to the control. Meanwhile, sulforaphane formation was significantly higher than that of sulforaphene; both reached the highest level at a UV-A intensity of 12 W, which was 3.89 and 3.37 times of that in the control, respectively.

### 2.7. Effect of UV-A Irradiation on α-Amylase Inhibition Rate in Broccoli and Radish Sprouts

**Figure 6:**
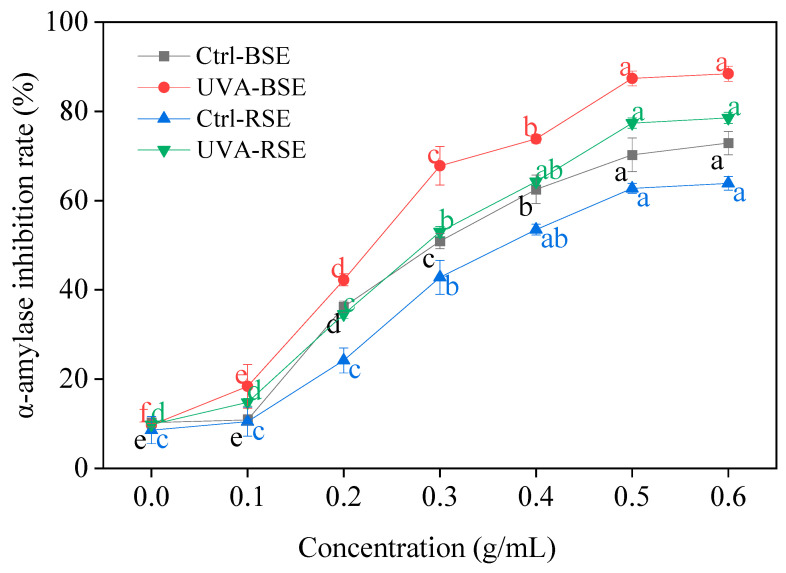
Effect of UV-A irradiation on α-amylase inhibition rate in broccoli and radish sprouts. Ctrl-BSE (black line): broccoli seeds treated with dark; UVA-BSE (red line): broccoli seeds treated with 12 W; Ctrl-RSE (blue line): radish seeds treated with dark; UVA-RSE (green line): radish seeds treated with 12 W. 0: water; 0.1, 0.2, 0.3, 0.4, 0.5, 0.6: 0.2, 0.4, 0.6, 0.8, 1.0, 1.2 g sprouts treated with 2 mL water, respectively. The letters of a–f are significantly different at *p <* 0.05.

According to Figure 6, it was easy to see that UV-A irradiation showed a positive effect on the α-amylase activity inhibition rate between the two kinds of sprouts. As the concentration of the broccoli and radish sprouts increased, the α-amylase activity inhibition rate also increased. At a concentration of 0.6 g/mL, the inhibition rate of α-amylase activity in broccoli and radish sprouts under UV-A irradiation was 9.06 and 7.94 times in comparison with the control, respectively.

### 2.8. Effect of UV-A Irradiation on α-Glucosidase Inhibition Rate in Broccoli and Radish Sprouts

**Figure 7:**
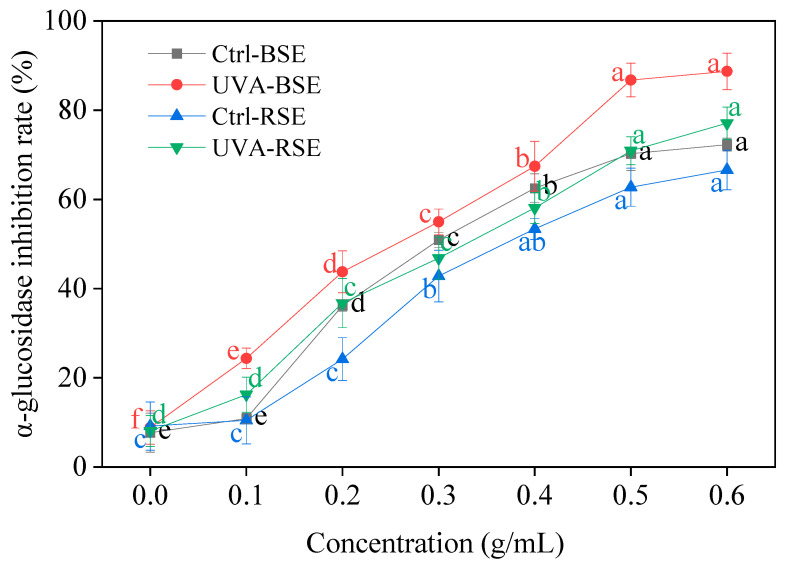
Effect of UV-A irradiation on α-glucosidase inhibition rate in broccoli and radish sprouts. Ctrl-BSE (black line): broccoli seeds treated with dark; UVA-BSE (red line): broccoli seeds treated with 12 W; Ctrl-RSE (blue line): radish seeds treated with dark; UVA-RSE (green line): radish seeds treated with 12 W. 0: water; 0.1, 0.2, 0.3, 0.4, 0.5, 0.6: 0.2, 0.4, 0.6, 0.8, 1.0, 1.2 g sprouts treated with 2 mL water, respectively. The letters of a–f are significantly different at *p* < 0.05.

With the sprout concentration increasing, the inhibitory rate of broccoli and radish sprouts against α-glucosidase enhanced continuously (Figure 7). The sprout concentration of 0.6 g/mL under UV-A irradiation resulted in the highest inhibition rate of α-glucosidase, which increased by 79.84% and 68.99% in comparison with the control, respectively.

### 2.9. Effect of UV-A Irradiation on Pancrelipase Inhibition Rate in Broccoli and Radish Sprouts

**Figure 8:**
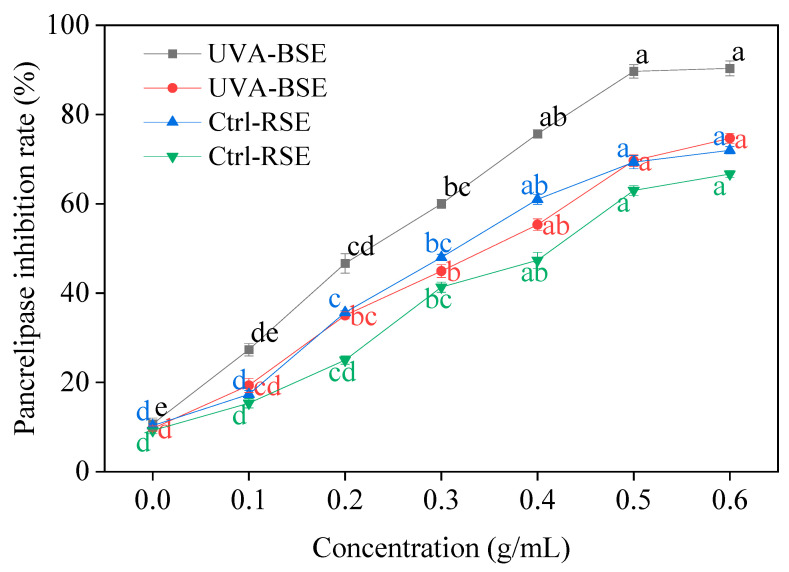
Effect of UV-A irradiation on pancrelipase inhibition rate in broccoli and radish sprouts. Ctrl-BSE (black line): broccoli seeds treated with dark; UVA-BSE (red line): broccoli seeds treated with 12 W; Ctrl-RSE (blue line): radish seeds treated with dark; UVA-RSE (green line): radish seeds treated with 12 W. 0: water; 0.1, 0.2, 0.3, 0.4, 0.5, 0.6: 0.2, 0.4, 0.6, 0.8, 1.0, 1.2 g sprouts treated with 2 mL water, respectively. The letters of a–e are significantly different at *p <* 0.05.

UV-A irradiation-treated broccoli and radish sprouts showed significant pancrelipase inhibitory activity (Figure 8). At a concentration of 0.6 g/mL, the inhibition rate of broccoli sprouts on pancrelipase activity was up to 90.31%, and that of radish sprouts was 74.42%, which increased by 79.84% and 64.74%, compared to the control, respectively.

## 3. Discussion

Broccoli and radish sprouts as new functional vegetables have attracted widespread attention from scholars all around the world. It has been reported that light plays an important role in plant growth, morphology and development. Tsormpatsidis et al. [14] reported that UV-A irradiation reduced the height of lettuce sprouts, while it increased secondary metabolites. In addition, the inhibition of stem elongation by UV-A irradiation has been confirmed in several plants, such as kale and basil [15,16]. This is consistent with the results of the present study, in which UV-A irradiation reduced the sprout length and weight of the broccoli and radish sprouts, but the radish sprouts were significantly higher than the broccoli sprouts; which might be due to photosynthetic characteristics and auxin levels among different UV-A intensities during germination [17].

In addition to growth, UV-A irradiation also regulates the accumulation of anthocyanin and chlorophyll in plants. According to Zhang et al. [18], UV-A irradiation significantly enhanced anthocyanin accumulation in radish sprouts by upregulating the expression of genes related to anthocyanin biosynthesis, including CRY, PHOT and COP1. Moreover, Nguyen et al. [19] also reported UV-A irradiation increased anthocyanin accumulation in “green and red perilla”. Similar results were also obtained in the present study. The anthocyanin content in broccoli and radish sprouts was enhanced after UV-A irradiation, and the likely explanation for this is the fact that the expression of genes and enzyme activity are related to anthocyanin biosynthesis under UV-A irradiation. Chlorophyll is an important product of photosynthesis. The results of Johnson et al. [20] showed that in the chlorophyll UV-absorption spectra, the absorbance of chlorophyll a at 340 and 389 nm was 51% and 72% of the absorbance peak in Sorghum bicolor, respectively. A previous study reported that the chlorophyll content in *Rosa hybrida* and *Fuchsia hybrida* was enhanced slightly by UV-A irradiation [21]. It is obvious that a similar phenomenon was detected in the present study. UV-A irradiation increased the chlorophyll content in the broccoli and radish sprouts, which might mean that the two kinds of sprouts directly absorbed specific wavelengths of UV-A, which then promoted the electron transport chain in the photosynthetic system, and thus promoted photosynthesis to produce more chlorophyll [22].

Plants produce soluble sugars during photosynthesis, which are the material basis for the synthesis of macromolecular compounds, such as polysaccharides and fats, and which also play an important role in participating in the carbon metabolism of plants. Mao et al. [22] found that the soluble sugar content in red and green pak choi was decreased after different UV-A intensities treatments. On the contrary, in the present study, the soluble sugar content in broccoli and radish sprouts increased significantly with the increasing of UV-A intensity. This might be due to high UV-A irradiation possibly damaging the integrity of the plant tissue, and thus hindering the normal metabolism of carbohydrates in the plant body [23]. However, this discrepancy needs further study. Soluble protein is one of the main components of plant cells and plays an important physiological function. No significant effect on soluble protein content under different UV-A intensities was observed in broccoli sprouts; however, in the present study it was found to be enhanced in radish sprouts. Interestingly, the phenomenon was similar to Tezuk [24], where UV-A irradiation was useful in promoting the accumulation of soluble protein content in radish plants. The likely explanation for this was that the soluble protein content might be related to the growth speed of two kinds of sprouts, but the exact mechanism needs to be further investigated.

Phenolic compounds and ascorbic acid play an important role in the antioxidant process of plants. Galactono-lactone dehydrogenase is a key enzyme in the ascorbic acid metabolic pathway, especially in the germination stage of the embryo [25]. Jia et al. [26] found that UV-A irradiation significantly increased the expression of ascorbic acid biosynthesis genes such as GME, VTC2, VTC4, GDH and GLDH levels in bean sprouts, thus significantly enhancing the ascorbic acid content. In the present study, the changes of ascorbic acid showed dramatic improvement in the broccoli and radish sprouts under UV-A irradiation, implying that UV-A irradiation also enhanced the ascorbic acid content by elevating the expression level and enzyme activity of the ascorbic acid biogenesis of two kinds of sprouts. Phenolic compound synthesis is influenced by multiple metabolic pathways [27]. A previous study reported that UV-A irradiation enhanced the phenolic compound content and PAL enzyme activity of sweet basil, especially with an intensity of 20 W/m^2^, resulting in a PAL enzyme activity and phenolic compound content of 38.4% and 36.3% higher than that of the control, respectively [8]. In the present study, the phenolic compound content in the two kinds of sprouts increased significantly under UV-A irradiation. The possible explanation for this was that UV-A radiation triggered oxidative stress and disrupted reactive oxygen homeostasis, thereby allowing phenolic synthases to function in stress-induced defense reactions that were directly involved in phenolic compound biogenesis [28].

UV-A as a signal could simultaneously stimulate different metabolic networks, and in the present study it also regulated glucosinolate metabolism in broccoli and radish sprouts. Zhuang et al. [29] reported that yellow and purple LED lights induced the glucoraphanin accumulation of broccoli sprouts by upregulating aliphatic glucosinolate biosynthetic genes, especially CYP79F1, CYP83A1, UGT74B1 and FMO_GS-OX1_. In addition, UV-A irradiation significantly increased the aliphatic glucosinolate content of Chinese kale by upregulating the expression of glucosinolate biosynthesis-related genes, including BCAT4, GGP1, SUR1, AOP2 and AOP3 [30]. Meanwhile, UV-A irradiation could also enhance the indolic glucosinolate accumulation of kale [10]. In the present study, the aliphatic and indolic glucosinolate content in broccoli and radish sprouts showed dramatic enhancement after UV-A irradiation. This might be because UV-A irradiation could induce gene expression in aliphatic and indole glucosinolate biosynthesis. Nevertheless, the specific mechanism of aliphatic and indole glucosinolate biosynthesis needs further study.

Sulforaphane and sulforaphene are the abundant isothiocyanates in broccoli and radish sprouts, respectively; which are the products formed by glucoraphanin and glucoraphenin under the catalysis of myrosinase. A previous study demonstrated that blue, purple and red light were beneficial to sulforaphane formation in broccoli florets [31]. O’Hare et al. [6] reported that the presence of ESP led to the hydrolysis of glucosinolate toward nitrile and thus decreased the isothiocyanate formation; broccoli sprouts contain ESP, but radish sprouts have no ESP. Therefore, in the present study, only 33.73% of the glucoraphanin translated to sulforaphane, but 77.99% of the glucoraphanin translated to sulforaphane. In addition to the glucosinolate content, myrosinase activity is also related to isothiocyanate formation. In the present study, myrosinase activity did not change or decrease after UV-A irradiation. Hence, the enhancement of sulforaphane and sulforaphene formation after UV-A irradiation was attributed to the increased content of glucoraphanin and glucoraphenin, rather than myrosinase activity.

α-amylase, α-glucosidase and pancrelipase are able to promote the breakdown of food in the mouth, stomach and intestines, releasing glucose into the bloodstream and raising blood sugar levels; thus, restraining the enzyme activity can predict the potential for the treatment of various disease such as diabetes [32,33]. The current approach to control hyperglycemia is to inhibit the activity of α-amylase, α-glucosidase and pancrelipase, thereby slowing down the digestion of carbohydrates in resorbable monosaccharides. In the present study, what was noteworthy was that UV-A irradiation showed excellent α-amylase, α-glucosidase and pancrelipase enzyme inhibition activity, and its effect on broccoli sprouts was better than that on radish sprouts. Therefore, two kinds of sprouts treated with UV-A radiation had the potential to be used as functional food and raw materials to reduce blood sugar.

## 4. Materials and Methods

### 4.1. Plant Materials, Cultivation and Treatment

Broccoli (*Brassica oleracea* L.) and radish (*Raphanus sativus* L.) seeds were purchased from Weifang Shouhe Seed Co., (Shandong, China). The varieties of broccoli were “Excellent broccoli”, and the varieties of radish were “Weixian radish”. They were disinfected with sodium hypochlorite and rinsed to neutral, then soaked in a water bath at 30 °C for 4 h. After that, the seeds were evenly spread in vermiculite-filled incubation trays at a density of 100 seeds per germination tray (60 cm × 30 cm × 4 cm), then germinated in the dark at 25 °C for 1 d [34,35] and incubated in UV-A incubators with different intensities of UV light (0, 4, 8, 12, 16 W) with a 16/8 h light/dark photoperiod. The model number of UV-A was F4T5BL and it was purchased from Beijing Ruilaixing Technology Co., Ltd., (Beijing, China). The plants were sprayed with deionized water twice a day, and samples were harvested at 5 d for measurement.

### 4.2. Determination of Sprout Length, Weight, Anthocyanin and Chlorophyll Content

A sample of 20 fresh sprouts was randomly selected from the sprouts treated with different UV light intensities for measuring sprout length and weight.

The content of anthocyanin was measured according to the method of Su et al. [36]. A total of 0.2 g of fresh sample was homogenized with 4 mL acidified ethanol. The homogenates were centrifuged at 10,000× *g* for 15 min, the supernatants were collected and the absorbance was measured at 535 nm.

The chlorophyll content was determined according to a previously published method [11]. Approximately 0.5 g of fresh sprouts were homogenized with 10 mL of 95% ethanol, and the homogenates were then filtered and transferred to a 25 mL volumetric flask. The absorbance measured at the wavelengths of 665 and 649 nm were chlorophyll a and chlorophyll b, respectively. The chlorophyll content in this study was the sum of chlorophyll a and chlorophyll b. The units of anthocyanin and chlorophyll content were expressed as mg/100 g FW.

### 4.3. Soluble Sugar and Soluble Protein Content Determination

The measurement of soluble sugar and soluble protein content was performed according to the method described by He et al. [11], though with some modifications. A total of 0.5 g of fresh sprouts were homogenized with 15 mL distilled water and then boiled for 20 min. The homogenates were allowed to cool and filter, then 1 mL of filtrate was taken and diluted by adding 6 mL distilled water. After that, 5 mL of anthrone solution was added to the extract and reacted in a boiling water bath for 10 min; finally, the absorbance value was measured at a 620 nm wavelength.

Approximately 0.2 g of fresh sprouts were ground into a homogenate using 4 mL of distilled water, then centrifuged at 10,000× *g* for 5 min. The supernatant was added to 5 mL of G-250 Coomassie brilliant blue solution and the reaction was carried out at room temperature for 5 min. The absorbance value was measured at a 595 nm wavelength. The soluble sugar and soluble protein content were expressed as mg/g FW.

### 4.4. Ascorbic Acid and Total Phenols Content Analysis

The method for determination of ascorbic acid content was referenced by Guo et al. [37], with some modifications. Approximately 0.3 g of fresh sprouts were extracted by grinding with 5 mL of 2% oxalic acid, and the homogenates were centrifuged at 8000× *g* for 5 min. The supernatant was filtered through a 0.45 μm membrane and then analyzed by HPLC.

A previously described method for total phenol content analysis was used in this study [37]. Fresh broccoli and radish sprouts (200 mg) were homogenized with 5 mL of 50% methanol, which was then centrifuged at 10,000× *g* for 15 min at room temperature. The supernatant was mixed with 1 mL Folin’s phenol solution (0.2 mol/L) and 2 mL Na_2_CO_3_ solution (2 g/100 mL), and the absorbance was measured at 765 nm after the reaction for 1.5 h at room temperature in the dark. The results of ascorbic acid and total phenol content were expressed as mg/100 g FW.

### 4.5. Glucosinolate Content Determination

The extraction and determination of glucosinolates were conducted by Guo et al. [38]. Approximately 0.5 g of fresh sprouts were homogenized with 4 mL of boiled 75% methanol solution and extracted at 80 °C for 15 min. After the homogenates were centrifuged at 10,000× *g* for 10 min, the supernatant was flowed through DEAE Sephadex A-25 column to purity glucosinolates, then analyzed by HPLC system with a UV detection at 226 nm. The unit of glucosinolate content was expressed as μmol/g FW.

### 4.6. Myrosinase Activity Assay

Determination was performed according to the experimental method of Guo et al. [37]. Fresh broccoli and radish sprouts (500 mg) were homogenized with 3 mL of 0.1 mol/L phosphate buffer (pH 6.5) under ice bath conditions, and centrifuged at 10,000× *g* for 15 min at 4 °C. The supernatant obtained was the crude enzyme. The crude enzyme and sinigrin (1 mmol/L) were mixed thoroughly, then reacted in a constant temperature water bath at 37 °C for 10 min. The enzyme was inactivated at 100 °C for 5 min and then measured using a glucose kit. One unit of enzyme activity was taken as 1 nmol of glucose per minute. The unit of myrosinase activity was expressed as U/g FW.

### 4.7. Sulforaphane and Sulforaphene Assay

The determination of sulforaphane and sulforaphene was referred to Guo et al. [39]. Approximately 0.5 g of frozen broccoli and radish sprouts were ground and mixed with 4 mL distilled water, then added to 4 mL dichloromethane. The dichloromethane phase was evaporated and dissolved with 2 mL of 10% acetonitrile, then analyzed by HPLC (Thermo Fisher Scientific Dionex Ultimate 3000, Thermo Field Biological Co. Ltd., Chelmsford, MA, USA). The units of sulforaphane and sulforaphene formation were expressed as mg/100 g FW.

### 4.8. Hypoglycemia-Related Enzyme Activity Assay

#### 4.8.1. Sample Treatments

Fresh broccoli and radish sprouts (0, 0.1, 0.2, 0.3, 0.4, 0.5 and 0.6 g) under 12 W irradiation were homogenized with 4 mL 50% ethanol, then the homogenates were concentrated with a nitrogen blow-drying apparatus, and 2 mL distilled water was added to re-dissolve the homogenates.

#### 4.8.2. α-Amylase Inhibitory Rate

The α-amylase inhibitory activity was determined by reference to Chen et al. [40], with slight modifications. The α-amylase solution (1 U/mL) and different concentrations of sample solution were added into a test tube, incubated for 10 min in a water bath at 37 °C, and finally, 1 mL DNS reagent was added. The enzyme was inactivated in a boiling water bath for 5 min, cooled and diluted by adding 5 mL of distilled water, then the absorbance value was measured at 520 nm. The α-amylase inhibitory rate was calculated using the following equation:

$$R/\%=\left(1-\frac{{OD}_{A}-{OD}_{a}}{{OD}_{B}-{OD}_{b}}\right)\times 100\%$$

where *R* is α-amylase inhibitory activity, %; *OD_A_* is the absorption value of the sample, enzyme and substrate; *ODa* is the absorption value of the sample, buffer and substrate; *OD_B_* is the absorption value of the buffer, enzyme and substrate; *ODb* is the absorption value of the buffer, buffer and substrate.

#### 4.8.3. α-Glucosidase Inhibitory Rate

The determination of α-glucosidase inhibitory activity was slightly adjusted with the method of Ke et al. [41]. The α-glucosidase solution (0.5 U/mL) and different concentrations of the sample solution were incubated in a water bath at 37 °C for 15 min, before 0.5 mL of PNPG solution was added as substrate and finally 5 mL of 0.1 mol/L Na_2_CO_3_ was added to terminate the reaction. The absorbance value was recorded at 400 nm and the calculation method was consistent with α-amylase inhibitory activity.

#### 4.8.4. Pancreatic Lipase Inhibitory Rate

A previously described method for pancreatic lipase inhibitory activity was used in this study [42]. The sprouts were treated as above. The sample solution of different concentrations, pancreatic lipase solution and Tris-HCL buffer solution were mixed, incubated at 37 °C for 10 min, before 6 mL 4-nitrophenylaurate was added as the substrate. The solution was reacted at 37 °C for 20 min and terminated in a boiling water bath for 10 min. The absorbance value was measured at 405 nm, and the calculation method was consistent with α-amylase inhibitory activity.

### 4.9. Statistical Analyses

All data are presented as the mean ± standard deviation of three repeats. The data were statistically analyzed with SPSS 26 (SPSS Inc., Chicago, IL, USA) and mapped with Origin 2021. Data were statistically analyzed by one-way ANOVA and Duncan’s multiple-range tests. Differences at *p* < 0.05 were considered significant.

## 5. Conclusions

In conclusion, our results acquired in the present study demonstrated that UV-A irradiation reduced the growth of broccoli and radish sprouts. Nevertheless, UV-A irradiation, especially 12 W of UV-A irradiation, significantly induced health compound accumulation, including anthocyanin, chlorophyll, polyphenol and ascorbic acid. Furthermore, 12 W of UV-A intensity treatment also resulted in the highest aliphatic and indole glucosinolate content, especially glucoraphanin and glucoraphenin, and contributed to the increase of sulforaphane and sulforaphane formation, respectively. On the other hand, broccoli and radish sprouts treated with UV-A irradiation of 12 W showed a significant inhibitory effect on α-amylase, α-glucosidase and pancrelipase, and the broccoli sprouts were even better. Hence, UV-A irradiation under 12 W might be applied to improve the quality of broccoli and radish sprouts so as to achieve the purpose of alleviating diabetes. In addition, the next study will compare the hypoglycemic activity of the two kinds of sprouts in vivo to provide a basis for the application of the sprouts.

## Figures and Tables

**Table 1:** Effect of UV-A irradiation on glucosinolate content in broccoli and radish sprouts. BSE: broccoli sprout extracts; RSE: radish sprout extracts. 0 W: seeds treated with dark; 4 W, 8 W, 12 W, 16W: seeds treated with UV-A, respectively. GRA: Glucoraphanin; RAE: Glucoraphenin; GNA: Gluconapin; GER: Glucoerucin; RSA: Glucoraphasatin; PRO: Progoitrin; 4HGB: 4-Hydroxyglucobrassicin; GB: Glucobrassicin; 4MGB: 4-Methoxyglucobrassicin; NGB: Neoglucobrassicin. The letters of a–e are significantly different at *p <* 0.05.

		μmol/g										
Light Intensity/W		Aliphatic Glucosinolate						Indole Glucosinolate				Total Glucosinolate
		GRA	RAE	GNA	GER	RSA	PRO	4HGB	GB	4MGB	NGB	
BSE	0	3.61 ± 0.07 ^e^	—	0.99 ± 0.01 ^e^	0.35 ± 0.02 ^e^	—	—	0.15 ± 0.01 ^d^	0.36 ± 0.03 ^e^	0.51 ± 0.01 ^d^	0.11 ± 0.01 ^e^	6.08 ± 0.16 ^e^
	4	5.86 ± 0.21 ^d^	—	1.37 ± 0.01 ^d^	0.52 ± 0.01 ^d^	—	—	0.26 ± 0.02 ^c^	0.39 ± 0.02 ^d^	0.52 ± 0.02 ^c^	0.15 ± 0.02 ^d^	9.07 ± 0.31 ^d^
	8	8.31 ± 0.28 ^c^	—	1.73 ± 0.01 ^c^	0.74 ± 0.00 ^c^	—	—	0.27 ± 0.02 ^b^	0.34 ± 0.04 ^c^	0.53 ± 0.01 ^b^	0.17 ± 0.02 ^c^	12.09 ± 0.38 ^c^
	12	10.83 ± 0.19 ^a^	—	2.17 ± 0.02 ^a^	1.17 ± 0.01 ^a^	—	—	0.33 ± 0.01 ^a^	0.40 ± 0.04 ^b^	0.54 ± 0.01 ^a^	0.21 ± 0.05 ^a^	15.62 ± 0.33 ^a^
	16	9.78 ± 0.21 ^b^	—	1.86 ± 0.02 ^b^	1.05 ± 0.01 ^b^	—	—	0.33 ± 0.00 ^a^	0.42 ± 0.02 ^a^	0.54 ± 0.01 ^a^	0.18 ± 0.03 ^b^	14.16 ± 0.30 ^b^
RSE	0	—	1.47 ± 0.03 ^e^	0.59 ± 0.01 ^e^	—	0.16 ± 0.03 ^e^	0.22 ± 0.02 ^c^	0.05 ± 0.00 ^c^	0.22 ± 0.04 ^d^	0.31 ± 0.01 ^b^	—	3.02 ± 0.14 ^e^
	4	—	1.89 ± 0.04 ^d^	0.68 ± 0.01 ^d^	—	0.25 ± 0.02 ^d^	0.25 ± 0.04 ^b^	0.08 ± 0.01 ^b^	0.23 ± 0.04 ^c^	0.31 ± 0.01 ^b^	—	3.69 ± 0.17 ^d^
	8	—	2.78 ± 0.04 ^c^	0.75 ± 0.00 ^c^	—	0.33 ± 0.02 ^c^	0.27 ± 0.02 ^a^	0.08 ± 0.00 ^b^	0.24 ± 0.04 ^b^	0.31 ± 0.02 ^b^	—	4.76 ± 0.14 ^c^
	12	—	4.18 ± 0.07 ^a^	1.04 ± 0.03 ^a^	—	0.56 ± 0.04 ^a^	0.19 ± 0.01 ^d^	0.09 ± 0.00 ^a^	0.25 ± 0.04 ^a^	0.32 ± 0.01 ^a^	—	6.63 ± 0.20 ^a^
	16	—	3.92 ± 0.07 ^b^	0.82 ± 0.02 ^b^	—	0.45 ± 0.05 ^b^	0.19 ± 0.02 ^d^	0.08 ± 0.02 ^b^	0.24 ± 0.00 ^b^	0.32 ± 0.02 ^a^	—	6.02 ± 0.20 ^b^

## Data Availability

The data is contained within the manuscript.

## References

[1] Bousquet J., Le Moing V., Blain H., Czarlewski W., Zuberbier T., de la Torre R., Pizarro Lozano N., Reynes J., Bedbrook A., Cristol J.P. (2021). Efficacy of broccoli and glucoraphanin in COVID-19: From hypothesis to proof-of-concept with three experimental clinical cases. World Allergy Organ. J..

[2] Patel B., Mann G.E., Chapple S.J. (2018). Concerted redox modulation by sulforaphane alleviates diabetes and cardiometabolic syndrome. Free Radic. Biol. Med..

[3] Xing J.J., Cheng Y.L., Chen P., Shan L., Ruan R., Li D., Wang L.J. (2018). Effect of high-pressure homogenization on the extraction of sulforaphane from broccoli (*Brassica oleracea*) seeds. Powder Technol..

[4] Baenas N., Gómez-Jodar I., Moreno D.A., García-Viguera C., Periago P.M. (2017). Broccoli and radish sprouts are safe and rich in bioactive phytochemicals. Postharvest Biol. Technol..

[5] Francis H., Debs E., Koubaa M., Alrayess Z., Maroun R.G., Louka N. (2022). Sprouts use as functional foods. optimization of germination of wheat (*Triticum aestivum L*.), alfalfa (*Medicago sativa L.*), and radish (*Raphanus sativus L.*) seeds based on their nutritional content evolution. Foods.

[6] O’Hare T.J., Williams D.J., Zhang B., Wong L.S., Jarrett S., Pun S., Jorgensen W., Imsic M. (2009). Radish sprouts versus broccoli sprouts: A comparison of anti-cancer potential based on glucosinolate breakdown products. Acta Hortic..

[7] Ahammed G.J., Chen Y., Liu C.C., Yang Y.X. (2022). Light regulation of potassium in plants. Plant Physiol. Biochem..

[8] Kang S., Kim J.E., Zhen S., Kim J. (2022). Mild-Intensity UV-A radiation applied over a long duration can improve the growth and phenolic contents of sweet basil. Front. Plant Sci..

[9] Moreira-Rodríguez M., Nair V., Benavides J., Cisneros-Zevallos L., Jacobo-Velázquez D. (2017). UVA, UVB Light, and methyl jasmonate, alone or combined, redirect the biosynthesis of glucosinolates, phenolics, carotenoids, and chlorophylls in broccoli sprouts. Int. J. Mol. Sci..

[10] Jiang H., Li Y., He R., Tan J., Liu K., Chen Y., Liu H. (2022). Effect of supplemental UV-A intensity on growth and quality of kale under red and blue light. Int. J. Mol. Sci..

[11] He R., Zhang Y., Song S., Su W., Liu H. (2021). UV-A and FR irradiation improves growth and nutritional properties of lettuce grown in an artificial light plant factory. Food Chem..

[12] Farag M.A., Aboul Naser A.F., Zayed A., Sharaf El-Dine M.G. (2023). Comparative insights into four major legume sprouts efficacies for diabetes management and its complications: Untargeted versus targeted NMR biochemometrics approach. Metabolites.

[13] Laila O., Murtaza I., Muzamil S., Imtiyaz Ali S., Abid Ali S., Ahamad Paray B., Gulnaz A., Vladulescu C., Mansoor S. (2023). Enhancement of nutraceutical and anti-diabetic potential of fenugreek (*Trigonella foenum-graecum*). Sprouts with natural elicitors. Saudi Pharm. J..

[14] Tsormpatsidis E., Henbest R.G.C., Davis F.J., Battey N.H., Hadley P., Wagstaffe A. (2008). UV irradiance as a major influence on growth, development and secondary products of commercial importance in Lollo Rosso lettuce ‘Revolution’ grown under polyethylene films. Environ. Exp. Bot..

[15] Choi D.-S., Nguyen T.K.L., Oh M.-M. (2022). Growth and biochemical responses of kale to supplementary irradiation with different peak wavelengths of UV-A light-emitting diodes. Hortic. Environ. Biotechnol..

[16] Qian M., Kalbina I., Rosenqvist E., Jansen M.A.K., Strid Å. (2023). Supplementary UV-A and UV-B radiation differentially regulate morphology in ocimum basilicum. Photochem. Photobiol. Sci..

[17] de Wit M., Galvão V.C., Fankhauser C. (2016). Light-mediated hormonal regulation of plant growth and development. Annu. Rev. Plant Biol..

[18] Zhang X., Su N., Jia L., Tian J., Li H., Huang L., Shen Z., Cui J. (2018). Transcriptome analysis of radish sprouts hypocotyls reveals the regulatory role of hydrogen-rich water in anthocyanin biosynthesis under UV-A. BMC Plant Biol..

[19] Nguyen L.T.K., Oh M.M. (2022). Growth and biochemical responses of green and red perilla supplementally subjected to UV-A and deep-blue LED lights. Photochem. Photobiol..

[20] Johnson G.A., Day T.A. (2010). Enhancement of photosynthesis in sorghum bicolor by ultraviolet radiation. Physiol. Plant..

[21] Helsper J.P.F.G., Ric de Vos C.H., Maas F.M., Jonker H.H., Van Den Broeck H.C., Jordi W., Pot C.S., Keizer L.C.P., Schapendonk A.H.C.M. (2013). Response of selected antioxidants and pigments in tissues of Rosa hybrida and Fuchsia hybrida to supplemental UV-A exposure. Physiol. Plant..

[22] Mao P., Duan F., Zheng Y., Yang Q. (2020). Blue and UV-A light wavelengths positively affected accumulation profiles of healthy compounds in Pak-choi. J. Sci. Food Agric..

[23] Lemoine R., Camera S.L., Atanassova R., Dédaldéchamp F., Allario T., Pourtau N., Bonnemain J.-L., Laloi M., Coutos-Thévenot P., Maurousset L. (2013). Source-to-sink transport of sugar and regulation by environmental factors. Front. Plant Sci..

[24] Tezuka T., Yamaguchi F., Ando Y. (1994). Physiological activation in radish plants by UV-A radiation. J. Photochem. Photobiol. B Biol..

[25] Xu M.-J., Dong J.-F., Zhu M.-Y. (2005). Effects of germination conditions on ascorbic acid level and yield of soybean sprouts. J. Sci. Food Agric..

[26] Jia L., Tian J., Wei S., Zhang X., Xu X., Shen Z., Shen W., Cui J. (2017). Hydrogen gas mediates ascorbic acid accumulation and antioxidant system enhancement in soybean sprouts under UV-A irradiation. Sci. Rep..

[27] Sun Y., Luo M., Ge W., Zhou X., Zhou Q., Wei B., Cheng S., Ji S. (2022). Phenylpropanoid metabolism in relation to peel browning development of cold-stored ‘Nanguo’ pears. Plant Sci..

[28] Hideg É., Jansen M.A.K., Strid K. (2013). UV-B exposure, ROS, and stress: Inseparable companions or loosely linked associates?. Trends Plant Sci..

[29] Zhuang L., Huang G., Li X., Xiao J., Guo L. (2022). Effect of different LED lights on aliphatic glucosinolates metabolism and biochemical characteristics in broccoli sprouts. Food Res. Int..

[30] He R., Li Y., Ou S., Gao M., Zhang Y., Song S., Liu H. (2022). Regulation of growth and main health-promoting compounds of Chinese kale baby-leaf by UV-A and FR light. Front. Plant Sci..

[31] Xie C., Tang J., Xiao J., Geng X., Guo L. (2022). Purple light-emitting diode (LED) lights controls chlorophyll degradation and enhances nutraceutical quality of postharvest broccoli florets. Sci. Hortic..

[32] Bharadwaj R.P., Raju N.G., Chandrashekharaiah K.S. (2018). Purification and characterization of alpha-amylase inhibitor from the seeds of underutilized legume, mucuna pruriens. J. Food Biochem..

[33] Hossain U., Das A.K., Ghosh S., Sil P.C. (2020). An overview on the role of bioactive α-glucosidase inhibitors in ameliorating diabetic complications. Food Chem. Toxicol..

[34] Guo L., Yang R., Wang Z., Guo Q., Gu Z. (2014). Glucoraphanin, sulforaphane and myrosinase activity in germinating broccoli sprouts as affected by growth temperature and plant organs. J. Funct. Foods.

[35] Jia L., Wang T., Sun Y., Zhang M., Tian J., Chen H., Shen Z., Abro H., Su N., Cui J. (2019). Protective effect of selenium-enriched red radish sprouts on carbon tetrachloride-induced liver injury in mice. J. Food Sci..

[36] Su N., Liu Z., Wang L., Liu Y., Niu M., Chen X., Cui J. (2022). Improving the anthocyanin accumulation of hypocotyls in radish sprouts by hemin-induced NO. BMC Plant Biol..

[37] Guo L., Yang R., Wang Z., Guo Q., Gu Z. (2014). Effect of NaCl stress on health-promoting compounds and antioxidant activity in the sprouts of three broccoli cultivars. Int. J. Food Sci. Nutr..

[38] Guo L., Yang R., Gu Z. (2016). Cloning of genes related to aliphatic glucosinolate metabolism and the mechanism of sulforaphane accumulation in broccoli sprouts under jasmonic acid treatment. J. Sci. Food Agric..

[39] Guo L., Yang R., Wang Z., Gu Z. (2015). Effect of freezing methods on sulforaphane formation in broccoli sprouts. RSC Adv..

[40] Chen C., Zhang B., Huang Q., Fu X., Liu R.H. (2017). Microwave-assisted extraction of polysaccharides from moringa oleifera Lam. leaves: Characterization and hypoglycemic activity. Ind. Crops Prod..

[41] Ke S., Wei B., Qiu W., Zhou T., Wang S., Chen J., Chen J., Zhang H., Jin W., Wang H. (2020). Structural characterization and alpha-glucosidase inhibitory and antioxidant activities of fucoidans extracted from saccharina japonica. Chem. Biodivers..

[42] Ercan P., El S.N. (2016). Inhibitory effects of chickpea and Tribulus terrestris on lipase, alpha-amylase and alpha-glucosidase. Food Chem..

